# Factors affecting length of stay in forensic hospital setting: need for therapeutic security and course of admission

**DOI:** 10.1186/s12888-015-0686-4

**Published:** 2015-11-23

**Authors:** Mary Davoren, Orla Byrne, Paul O’Connell, Helen O’Neill, Ken O’Reilly, Harry G. Kennedy

**Affiliations:** Department of Psychiatry, Trinity College Dublin, Dublin, Ireland; National Forensic Mental Health Service, Central Mental Hospital, Dundrum, Dublin 14 Ireland

**Keywords:** Forensic, Hospital, Length of stay, Prediction, DUNDRUM toolkit

## Abstract

**Background:**

Patients admitted to a secure forensic hospital are at risk of a long hospital stay. Forensic hospital beds are a scarce and expensive resource and ability to identify the factors predicting length of stay at time of admission would be beneficial. The DUNDRUM-1 triage security scale and DUNDRUM-2 triage urgency scale are designed to assess need for therapeutic security and urgency of that need while the HCR-20 predicts risk of violence. We hypothesized that items on the DUNDRUM-1 and DUNDRUM-2 scales, rated at the time of pre-admission assessment, would predict length of stay in a medium secure forensic hospital setting.

**Methods:**

This is a prospective study. All admissions to a medium secure forensic hospital setting were collated over a 54 month period (*n* = 279) and followed up for a total of 66 months. Each patient was rated using the DUNDRUM-1 triage security scale and DUNDRUM-2 triage urgency scale as part of a pre-admission assessment (*n* = 279) and HCR-20 within 2 weeks of admission (*n* = 187). Episodes of harm to self, harm to others and episodes of seclusion whilst an in-patient were collated. Date of discharge was noted for each individual.

**Results:**

Diagnosis at the time of pre-admission assessment (adjustment disorder v other diagnosis), predicted legal status (sentenced v mental health order) and items on the DUNDRUM-1 triage security scale and the DUNDRUM-2 triage urgency scale, also rated at the time of pre-admission assessment, predicted length of stay in the forensic hospital setting. Need for seclusion following admission also predicted length of stay.

**Conclusions:**

These findings may form the basis for a structured professional judgment instrument, rated prior to or at time of admission, to assist in estimating length of stay for forensic patients. Such a tool would be useful to clinicians, service planners and commissioners given the high cost of secure psychiatric care.

## Background

There are two hazards facing a patient admitted to a secure forensic hospital. The first is detention at an inappropriately high level of therapeutic security, with restrictions and intrusions on freedom, privacy and choice out of proportion to what is needed for safe treatment. The second is an inappropriately long length of stay. The first of these problems can be addressed through the use of structured professional judgement instruments for assessing need of therapeutic security, such as the DUNDRUM-1 triage security instrument [1, 2] in the context of an appropriate governance structure [3]. The second can be addressed by ensuring that the individual care and treatment plan and the recovery pathway from higher to lower levels of therapeutic security and eventual restoration of autonomy is guided by treatment programmes relevant to needs derived from the seriousness [4, 5] and probability [6, 7] of the risks the person presents. This can be addressed using risk assessment and needs assessment instuments in combination with more specific assessments of treatment outcome and recovery [8, 9]. This paper set out to identify risk factors for longer lengths of stay in secure forensic hospital care, so that length of stay can be actively managed from the outset.

### Length of stay and policy initiatives

In 1997, the Department of Health UK identified managing length of stay as a key method of managing the efficiency of hospital services (Department of Health (UK), 1997). Between 2000 and 2003, the NHS UK developed a system of ‘payment by results’ which linked funding offered to hospitals directly to hospital efficiency and productivity [10–13]. Similar healthcare payment systems were already in place in other countries for example under Medicare in the United States the ‘prospective payment system’ had been in place since 1983 [14]. The concept of ‘payment by results’ was finally widely introduced in 2003 after the publication of “Reforming NHS financial flows” [13] and the response to the consultation in relation to that document [10–13]. The introduction of ‘payment by results’ financially incentivized hospitals to decrease length of stay for patients, as it linked the number of individuals treated with the amount of money hospitals were paid. This in turn led to an increased focus on factors affecting length of stay in hospital settings.

Farrar et al. compared length of stay in hospitals in England that had implemented ‘payment by results’ with hospitals in Scotland where that system did not exist and found that length of hospital stay decreased by 8 per 100 patient days more in the English hospital group than the control group [15]. In 1995 Belgium implemented a similar model to ‘payments by results’ however their model was even more closely linked to length of stay. Under the Belgian system each patient presentation is attributed a fixed number of hospital days and this is paid for prospectively at a standard rate per day with a lower payment given for any excess days; therefore much of the cost of longer stays is borne by the treating hospital [16]. Studies have shown that introducing these systems of payment for healthcare reduced length of hospital stay across a wide range of procedures and diagnoses in the UK, US and elsewhere [16–19].

The financially incentivized decrease in length of hospital stay caused some concern that patients may have been prematurely discharged leading to worse overall outcomes and poorer quality of care. The ‘Quicker but sicker’ study found that unstable patients were discharged earlier after the introduction of prospective payment systems in the U.S. [20]. Kossovsky et al. [21] in a study of Swiss patients admitted for treatment of congestive cardiac failure found that longer length of stay was significantly associated with improved quality of care and improved outcomes. Other studies in contrast, have shown no worsening in outcomes after introduction of prospective payment systems [22, 23].

Reduced length of hospital stay is associated with reduced costs; therefore it follows that the more expensive the hospital bed the greater the potential saving from reducing length of stay [24]. While secure forensic beds cost much less than for example cardiac telemetry beds, they are more expensive than general adult psychiatry admission beds so that effective management of length of stay in secure forensic hospital settings has the potential to yield cost savings. The Centre for Mental Health UK noted that secure forensic beds have high costs and treat a small number of patients [25]. The Report of the Schizophrenia Commission criticized length of stay in forensic hospital settings and stated that patients in secure care “Often stay too long in very expensive and often unsuitable provision” [26].

### Forensic hospitals and length of stay

Secure forensic psychiatry units have a dual purpose, to treat mental illness and also to treat offending behaviour [27]. Patients are admitted to secure forensic services at medium and high levels of therapeutic security because they have a history of serious violence and pose a serious or grave risk to the public. Care must be taken prior to discharging mental health patients with a history of serious violence [28, 29]. Therefore what is considered an appropriate length of hospital stay and an appropriate aftercare plan for a patient in a general adult psychiatry setting may not be appropriate for a patient in a forensic setting and care plans must be altered to give sufficient weight to histories of life threatening violence [30].

The Glancy Report stated in 1974 that after a maximum of eighteen months in a medium secure setting an alternative placement must be considered for a patient [31]. However studies in both Ireland and the UK have shown that length of stay in medium and high secure forensic hospitals is often much longer, with many patients staying longer than 5 years [32, 33]. Homicide inquiries, for example the Barrett Inquiry and the Inquiry into the care and treatment of Peter Bryan, have criticised shorter lengths of stay as contributory factors in serious adverse outcomes [34, 35].

Originally high secure forensic units were designed as ‘long stay’, medium secure forensic units as ‘medium stay’ and low secure units as ‘short stay’. Over time secure forensic services have developed long term medium secure and long term low secure units [36]. Changes in the availability of general adult psychiatric beds may lead to diversion of patients from adult psychiatric care pathways to forensic services and pathways [32, 37] while more specific availability of low acute low secure beds may prevent patients escalating to medium secure beds [38, 39]. Changes in admission and discharge policy over time and changes in bed numbers may have impacted on thresholds for admission [40]. These and other systemic factors may have as much impact on length of stay as clinical or legal factors [41].

Recent research has addressed risk factors for longer lengths of stay using cross-sectional methods [32, 36, 41, 42], case–control methods [43] and prospective approaches [44–46].

### Structured professional judgement and length of stay

Structured professional judgment instruments are designed to combine the best features of actuarial risk instruments by ensuring that all items that predict the outcome are considered, while still allowing the clinicians flexibility regarding the overall assessment on a case by case basis [47–52]. Excluding an actuarial element in the HCR-20 does not reduce the accuracy of the HCR-20 [48]. Structured professional judgment instruments aim to improve consistency and transparency in decision-making. The most widely known structured professional judgment instrument, The Historical Clinical Risk for Violence-20 (HCR-20), has been shown to predict violence in mentally disordered offenders [6, 7, 53, 54]. The goal of treatment in secure forensic hospital services is to reduce the risk of violence and to reduce the seriousness of the risk. Risk, the probability of violence, might be expected to predict length of stay but to our knowledge risk assessment instruments have not been studied as predictors or determinants of length of stay.

In forensic psychiatry structured professional judgment instruments exist to examine specific types of violence, such as the Manual for Sexual Violence Risk-20 (SVR-20) [50], risk of suicide for example the Suicide Risk Assessment and Management Manual (S-RAMM) [51], and need for therapeutic security for example DUNDRUM-1 triage security scale [1] and Health Of the Nation-Secure (HONOS-Secure) [55]. The DUNDRUM toolkit is a suite of five scales. The first two scales, the DUNDRUM-1 triage security scale and the DUNDRUM-2 triage urgency scale are designed to assess the need for therapeutic security and the urgency of that need respectively. Together they form the ‘preadmission scale’ of the DUNDRUM toolkit. They are different from risk assessment. They are designed to predict need for therapeutic security and the urgency of that need, and focus mainly on ‘seriousness’ of violence rather than risk of violence [2, 3].

The need for therapeutic security is based on judgements of seriousness of violence and related matters such as risk of absconding as much as or more than probability (risk). The need for therapeutic security at the time of admission might therefore be expected to predict length of stay. However to date there is no evidence on which to base a structured professional judgment instrument for length of stay.

Despite the limited evidence base examining the area of length of stay in forensic settings, an ability to predict length of stay at the time of admission would be of enormous benefit to patients and services. Any attempt to examine length of stay in forensic psychiatry settings must balance the potential for economic savings with decreased length of stay against the potential for serious adverse outcome in this high risk patient group if patients are prematurely discharged.

### Objectives

The aim of this study is to examine whether or not scores on the items of the DUNDRUM-1 triage security and the DUNDRUM-2 triage urgency scales rated at the time of pre-admission assessment, would predict length of stay in a forensic hospital setting as expected [41] based on cross-sectional data [32, 41] and prospective data [44]. Although a detailed assessment of risk at the time of admission or prior to admission is difficult, we also examined whether the items of the HCR-20, a well-validated assessment of risk of violence, could predict length of stay. We also set out to examine whether factors such as diagnosis or legal status might be predictive of length of stay. We used logistic regression modelling to assess the relative importance of any factors identified as predictors of length of stay.

## Methods

### Study design

This was a naturalistic prospective whole cohort study. All patients who were admitted from 1 January 2010 to 30 June 2014 a 54-month period, were observed and the observation period continued for a further 12 months until 1st July 2015, to ensure all patients had a minimum follow up period of at least 12 months.

### Setting

This study was set in the Central Mental Hospital, Dundrum (CMH). The Central Mental Hospital is the site of Ireland’s national forensic mental health service. It is the only secure hospital in the Republic of Ireland; it serves a population of 4.6 million and contains high, medium and low levels of therapeutic security on one site [56]. At the time of this study there were 94 secure forensic beds at high, medium and low security, or 2/100,000 population.

All patients referred for admission are assessed by a forensic psychiatrist and forensic community mental health nurse prior to being placed on the hospital waiting list. All such patients are also rated using the DUNDRUM-1 triage security and the DUNDRUM-2 triage urgency scales which together make up the DUNDRUM preadmission tool [57]. It has previously been shown that ratings on the DUNDRUM-1 triage security scale and the DUNDRUM-2 triage urgency scale predict need for therapeutic security and the urgency of that need [2, 3].

The start date of this study coincided with a reform of the Criminal Law (Insanity) Act so that patients found unfit to stand trial or not guilty by reason of insanity could be granted conditional discharge by the Mental Health Review Board, a statutory body that is independent in the exercise of its powers.

Once admitted, all patients in the Central Mental Hospital are detained either under the Mental Health Act 2001 (civil detention) or the Criminal Law (Insanity) Act 2006. The Central Mental Hospital Dundrum is the only centre in the Republic of Ireland designated under the Criminal Law (Insanity) Act to admit remand or sentenced prisoners, those found unfit to stand trial and persons found not guilty by reason of insanity. Under the Mental Health Act 2001, Ireland’s civil mental health legislation, the Central Mental Hospital accepts admissions of those patients who have been deemed to exceed the capacity of community general adult psychiatry admission units[58].

### Ethics

This study was approved by the research ethics, audit and effectiveness committee of the National Forensic Mental Health Service as a service evaluation project.

### Participants

Participants included all patients admitted to the Central Mental Hospital Dundrum between 1 January 2010 and 30 June 2014, a total enrollment period of 54 months. A total of 287 patients were admitted during the 54-month period, 239 (83.3 %) of whom were male. Patients were followed up until 1 July 2015, giving a total survey period of 66 months.

### Variables: outcome measures

The primary outcome measurement for this project was date of discharge from the secure hospital setting. Violent incidents following admission were recorded by a system of incident reporting combined with cross-checks by reference to patient’s notes, legal records of restraint and seclusion and central nurse management records. Seclusion, a legally sanctioned and controlled intervention used only when all other interventions to prevent violence have failed was recorded from legal registers.

### Variables: measurement instruments

Participants were assessed for need for therapeutic security and the urgency of that need, using the DUNDRUM-1 triage security and DUNDRUM-2 triage urgency scales.

The DUNDRUM-1 triage security scale is a structured professional judgment instrument designed to assist decision making when deciding the level of therapeutic security to which a patient should be admitted [57]. It consists of a scale of 11 items, as follows: seriousness of violence, seriousness of self-harm, immediacy of risk of violence, immediacy of risk of self-harm, specialist forensic need, absconding/eloping, preventing access, victim sensitivity and public confidence, complex risk of violence, institutional behaviour and legal process. Each item is scored zero to four, with each score tethered to a set of definitions. A patient rated mostly ‘4’ is likely to require high security, mostly ‘3’ will require medium security, mostly ‘2’ will require PICU, mostly ‘1’ is likely to be suitable for an open ward and mostly ‘0’ is likely to be manageable in the community setting. DUNDRUM-1 triage security items are not intended as a risk assessment instrument; rather they assess need for therapeutic security and are complementary to risk assessment instruments [57]. A mean score is calculated by dividing the sum of 11 items by 11, or the sum of nine items (omitting the two suicide related items) by nine. The mean DUNDRUM-1 score then has a range of 0 to 4 and is a guide to the levels above, for example a mean DUNDRUM-1 score between 2 and 3 is in keeping with medium secure need, a mean score above 3 suggests a need for high security.

The DUNDRUM-2 triage urgency scale is the second part of the DUNDRUM toolkit preadmission scale [57]. It is designed to assess the urgency of need for admission to a secure forensic hospital. It is intended to be used in conjunction with the DUNDRUM-1 triage security scale, however unlike the mainly static items of the DUNDRUM-1 triage security scale, the items of the DUNDRUM-2 triage urgency scale are designed to be dynamic [3, 57]. The scale consists of six items as follows: the first item is used to rate the patients current location, other items rate mental health, suicide prevention issues, humanitarian issues, systemic issues and legal urgency. The first item, which rates the patient’s urgency of need for admission based on their current location, takes into account a number of different possible locations a patient may be referred from for example a remand prison, a sentenced prison or a patient in a hospital at a lower level of security than is required or appropriate. In a similar way to the other scales of the DUNDRUM toolkit, each item is scored between ‘0’ and ‘4’, with each score tethered to a series of definitions in order to ensure reliability and accuracy of ratings. As it is a dynamic scale, it is intended that the DUNDRUM-2 triage urgency scale is repeated on a once weekly basis whilst a patient is on a waiting list for a secure forensic admission bed in order to best assess the patient's urgency of need on the waiting list. In a study of remand prisoners referred for admission to a medium secure forensic hospital setting, scores on the DUNDRUM-2 triage urgency scale predicted urgency of need for therapeutically secure beds [3].

Participants had a HCR-20 V2 completed within the first 2 weeks of admission in the majority of cases by their treating multi-disciplinary team, led by a consultant forensic psychiatrist. The HCR-20 is a structured professional judgement instrument to assist in the assessment and management of risk of violence [7, 47]. It consists of ten ‘historical’ items that are relatively fixed or stable over time. Item 7 is based on the psychopathy check list and is routinely omitted. Exclusion of the psychopathy item (H7) does not reduce the HCR-20’s accuracy [48]. There are also ten dynamic items that are sensitive to change. These include five ‘clinical’ or current items and five ‘risk management’ items concerning the near future. The ‘risk management’ items were rated for the conditional situations that the patient might remain in hospital and for the alternative that they might be discharged in the near future to whatever would be available in the community or prison at that time.

### Other variables

Other variables thought relevant to length of stay included gender, age, diagnosis, legal status on admission (remand pending trial, sentenced following conviction, unfit to stand trial, not guilty by reason of insanity (NGRI) or civilly detained and transferred from a non-forensic hospital). We also considered the final legal status as predicted at the time of admission (sentenced, unfit to stand trial, not guilty by reason of insanity (NGRI) and civil detention).

We considered the possibility that challenging behavior within the hospital might also influence length of stay, so data were gathered on acts of violence towards self and others and episodes of seclusion. This information was available from routine incident reporting and from statutory records of the use of seclusion.

### Study size

We completed a pseudo-pilot study using a cross-sectional sample of all patients in the hospital in March 2010. We showed that by dichotomizing DUNDRUM-1 Triage Security item 1 to compare those who scored ‘4’ against those who scored ‘3’ or less, with length of stay as the dependent variable, the effect size for difference in cross-sectional length of stay was Cohen’s d = 0.536 and this generated a 72.8 % chance of detecting a statistically significant difference at 5 % level in a sample of 95 in-patients.

### Statistical methods

All data were entered into SPSS version 22 [59]. No data were missing though HCR-20 data close to the time of admission were available only for 187 patients. We used Kaplan Meier survival analysis to ascertain which variables significantly distinguished subsequent length of stay (log-rank Mantel-Cox *X*^2^) and Cox regression (proportional hazards) for further modelling of factors influencing length of stay [59].

Kaplan-Meyer survival analysis is a method for examining the distribution of time between two events, in this study admission and discharge to and from a secure forensic hospital.^1^ The Cox ‘proportional hazards’ regression procedure is used for modelling time from admission to discharge (the ‘hazard’ in this study) based upon the values of given coordinates.^2^ These two tests generate median lengths of stay and standard errors of the median from which 95 % confidence intervals can be calculated. This is the most appropriate summary statistic for length of stay because of the nature of its statistical distribution as a survival function.

For risk assessment the receiver operating characteristic (ROC) area under the curve (AUC) was used and a significant result was taken as an AUC with a 95 % confidence interval that did not overlap 0.5, the line of ‘no information’.

## Results

### Participants

There were 279 admissions of 230 patients during the 54-month enrolment period of the study. Each admission had a pre-admission assessment completed by an assessing consultant forensic psychiatrist and forensic community mental health nurse. All 279 admissions had a pre-admission DUNDRUM-1 triage security scale and DUNDRUM-2 triage urgency scale completed prior to admission. Patients who had been on the waiting list for the Central Mental Hospital for some weeks prior to admission may have had several DUNDRUM-2 triage urgency scales completed during that time and therefore the most recent DUNDRUM-2 triage urgency scale that was completed prior to the date of admission was used for the purposes of this study.

The HCR-20 V2 was completed for 187 patients within the first 2 weeks of admission. The HCR-20 could not be completed within the first 2 weeks for the remaining 92 patients. The 92 for whom an early HCR-20 could not be obtained were not different in mean age (years 33.7 (10.2) vs 35.4 (10.7) ANOVA F = 1.6, *p* = 0.204) and following admission they were not more likely to be violent (*X*^2^ = 0.19, df = 1, *p* = 0.661), engage in self harm (*X*^2^ = 1.39, *p* = 0.259) or to be secluded (*X*^2^ = 1.7, *p* = 0.190) but they were more likely to be female (49 v 30 %, *X*^2^ = 6.5, *p* = 0.011), to have been admitted from prison (legal status *X*^2^ = 7.9, df = 3, *p* = 0.049) and to have adjustment reaction as their admission diagnosis (*X*^2^ = 10.6, df = 3, *p* = 0.014). They had lower mean DUNDRUM-1–9 item scores (2.3 (SD 0.6) vs 2.7 (0.6) F = 21.8, *p* < 0.001) and had shorter length of stay (median days 14.0 (SE 2.4) vs 90 (12.6) log rank Mantel Cox 143.7, *p* < 0.001).

### Descriptive data

There were 94 patients present in the hospital at the beginning of the study period. The majority of admissions during the study period, 232 (83.3 %), were male. The mean age at admission was 34.9 years, median 32.0 years (SD 10.5 years). There was no significant difference in age between the male and female patients (F = 0.01, df = 1, *p* = 0.911).

Table 1 shows the distribution of scores on the needs assessment and risk assessment instruments. The mean item score on the DUNDRUM-1 11 item scale, a measure of need for therapeutic security was 2.36 (SD 0.54) and on the DUNDRUM-1 nine item scale (omitting items TS2 and TS4 concerning suicide) the mean item score was 2.57 (SD0.60) in keeping with a group who exceed the low-secure threshold (2.0) for need of therapeutic security.

**Table 1:** Distribution of scores on needs assessment and risk assessment instruments

	Number	Mean	Median	Mode	SD	Min	Max
DUNDRUM-1_11 item mean score	279	2.36	2.45	2.45	0.54	0.91	3.73
DUNDRUM-1_9 item mean score	279	2.57	2.56	2.56	0.60	0.78	3.78
DUNDRUM-2 total score	279	11.7	12.0	13	2.93	4	20
HCR-20 H	187	13.2	14.0	16	3.47	3	18
HCR-20 C	187	6.3	7.0	8	2.73	0	10
HCR-20 R-in	187	4.9	5.0	3	2.49	0	10
HCR-20 R-out	187	8.7	9.0	10.0	1.63	0	10
HCR-20 total in	187	24.3	25.0	21.0	6.83	7	37
HCR-20 total out	187	28.1	30.0	33.0	6.47	7	38

For DUNDRUM 1_11, DUNDRUM 1_9 and DUNDRUM 2 mean scores, the range is 0–4; HCR-20 H range is 0–20; HCR-20 C and R range is 0–10; HCR-20 total range is 0–40

The mean item score on the 11 item DUNDRUM-1 scale for men (*n* = 232) was 2.38 (SD 0.52) and for women (*n* = 47) 2.24 (SD 0.59) ANOVA F = 2.94, df = 1, *p* = 0.088. For the nine-item DUNDRUM-1 the mean item score for men was 2.62 (SD 0.59) and for women 2.34 (SD 0.59) F = 8.88, *p* = 0.003.

Table 2 shows the distributions of length of stay for male and female patients, with women having significantly shorter lengths of stay. The main diagnosis influenced length of stay, with adjustment reaction having the shortest lengths of stay with increasing lengths of stay for affective disorders, schizophreniform psychoses and organic disorders (intellectual disability, autism spectrum disorders and acquired brain injury).

**Table 2:** Demographic variables, legal status and length of stay (days) to discharge or end of study. Calculated from Kaplan-Meyer survival analysis

	0			1			2			3			4			Log rank Mantel-Cox	
Item	*n*	Median	SE	*n*	Median	SE	*n*	Median	SE	*n*	Median	SE	*n*	Median	SE	*X* ^2^	*p*
Gender	Female			Male									Total				
	47	24.0	5.4	232	60.0	4.1							279	56.0	4.3	5.89	0.015
Primary diagnosis				Adjustment disorder			Bi-polar and affective			Schizophrenia spectrum			organic				
				35	14.0	5.4	43	41.0	7.0	186	67.0	5.9	15	79.0	79.8	28.4	0.001
Legal status on admission	Remand			Sentenced			Unfit to stand trial			Not guilty by reason of insanity			Civil transfer				
	122	55.0	6.9	127	53.0	4.0	9	12.0	0.8	13	1100.0	0.0	8	994.0	167.1	22.8	0.001
Legal status on admission	Prisoner (remand or sentenced)						Unfit to stand trial			Not guilty by reason of insanity			Civil transfer				
	249	55.0	3.6				9	12.0	0.8	13	1100.0	0.0	8	994.0	167.1	16.8	0.001
Predicted final legal status at time of admission	Sentence						Unfit to stand trial			Not guilty by reason of insanity			Civil transfer				
	214	46.0	3.7				15	90.0	54.1	42	1100.0	455.3	8	994.0	167.1	82.1	0.001
Final legal status (actual)	Sentenced						Unfit to stand trial			NGRI			Civil transfer				
	230	48.0	4.2				11	90.0	47.3	26	1100.0	0	8	994.0	167.1	80.0	0.001

Medians, standard errors and Log rank Mantel-Cox *X* ^2^, *n* = 279

Legal status on admission was a good predictor of length of stay. As there was no significant difference between remand and sentenced status, these were combined. The predicted legal status at the time of admission was as good a predictor of length of stay as the final legal status. By the end of the study period, final legal status had been decided for 275 of the patients (intra-class correlations admission to final 0.737, F = 6.6, *p* < 0.002; predicted to final ICC = 0.848, F = 12.2, *p* < 0.001), with the predicted outcomes tending to over-estimate the final numbers found unfit to stand trial and NGRI (Table 3). Predicted diagnosis was correct for 87 % overall. The greatest anomaly was for NGRI, with only 26 (62 %) being found NGRI of the 42 predicted.

**Table 3:** Legal status on admission, predicted final legal status (predicted at time of admission) and actual final legal status

	Legal status on admission	Final legal status as predicted at time of admission	Actual final legal status
Prison (sentenced)	127	214	230
Unfit to stand trial	9	15	11
Not guilty by reason of insanity	13	42	26
Civil	8	8	8
Remanded to prison (pre-trial, undecided)	122	0	4
Total	279	279	279

### Outcome data

At the end of the study period, 29 of those admitted remained as in-patients. The remainder of the patients in hospital at the end of the study were long term patients who had already been present in the hospital at the start of the study period.

### Main results

During the observation period, the mean length of stay for male patients was 304.3 days (Standard Error (SE) 38.3) compared with 202.6 days (SE 57.9) for females; median was 60 days for male patients and 24 days for female patients. Gender predicted length of stay, log-rank Mantel-Cox *X*^2^ = 5.89, df = 1, *p* = 0.015.

Diagnoses at the time of admission were divided into four categories, adjustment disorder (*n* = 38), schizophreniform disorder (*n* = 186), bi-polar and affective disorders (*n* = 43) and organic disorder, learning disability and autistic spectrum (*n* = 15). Note that ‘adjustment disorder’ included those with a diagnosis of personality disorder who were usually admitted because of an adjustment reaction with suicidal behaviour. Diagnosis at time of admission significantly predicted length of stay, log rank Mantel-Cox *X*^2^ = 28.4, df = 3, *p* < 0.001. Diagnosis was stable, with only 11 of 186 admission diagnoses of psychosis re-classified by the time of discharge as personality disorder or nil, 4 of 43 affective disorders reclassified as personality disorder or nil by the time of discharge and none of the 15 organic, learning disability and autistic spectrum disorders reclassified.

### Events during the admission and length of stay

Eleven (11) patients engaged in deliberate self-harm during the course of their admission. Deliberate self-harm during the course of the admission did not predict length of stay, log-rank Mantel-Cox *X*^2^ = 0.017, df = 4, *p* = 0.896.

Seventy-two patients engaged in violence towards others during the course of their admission. Episodes of harm to others did not predict length of stay, log-rank Mantel-Cox *X*^2^ = 2.073, df = 4, *p* = 0.150.

One-hundred-three patients required management in seclusion on at least one occasion during their admission. Requiring seclusion during admission did predict length of stay, log-rank Mantel-Cox *X*^2^ = 4.264, df = 4, *p* = 0.039. The median length of stay for patients who had at least one episode of seclusion during their admission was 64 days (SE 6.7) compared with 46 days (SE 6.4) for those patients who were not secluded.

### Scores on the DUNDRUM-1 triage security scale and length of stay

Table 4 shows that all but three of the items of the DUNDRUM-1 predicted length of stay. Those that did not predict length of stay were the two items concerning suicide and TS7 ‘preventing access’. Results are also given for the 187 for whom HCR-20 assessments were completed in the first 2 weeks of the admission. Although the significance was attenuated for most items, the same patterns remained except for ‘legal process’ which was no longer significant.

**Table 4:** DUNDRUM-1 triage security scale items and length of stay (days) to discharge or end of study

Score	0			1			2			3			4			Log rank Mantel-Cox *N* = 279		Log rank Mantel Cox *N* = 187	
Item	*n*	Median	SE	*n*	Median	SE	*n*	Median	SE	*n*	Median	SE	*n*	Median	SE	*X* ^2^	*P*	*X*2	*p*
TS1: seriousness of violence	7	12	3.9	36	18	6.5	47	49	5.5	92	54	6.9	97	78	15.7	39.9	0.001	17.9	0.001
TS2: seriousness of suicidal act	93	64	8.8	87	48	7.5	32	66	15.6	39	50	5.2	28	45	16.5	2.4	0.668	3.9	0.425
TS3: still in mental state that led to serious violence	11	12	1.1	19	26	13.8	84	49	5.9	49	76	9.5	116	67	9.9	20.7	0.001	9.5	0.049
TS4: still in mental state that led to serious suicidal act	97	67	13.5	44	41	16.6	86	55	4.6	16	64	26.0	36	49	2.2	2.02	0.733	4.1	0.389
TS5: specialist forensic need	15	34	23.8	73	34	6.4	73	49	6.6	78	102	25.4	40	67	15.0	27.3	0.001	17.6	0.001
TS6: absconding/eloping	5	49	41.6	25	14	5.9	123	42	5.5	103	79	19.4	23	61	25.6	25.1	0.001	13.3	0.01
TS7: preventing access	2	11	–	32	62	16.3	128	48	6.4	92	60	6.2	25	55	15.8	2.5	0.641	4.0	0.401
TS8: victim sensitivity & public confidence	16	66	36.0	60	26	6.6	84	50	5.9	68	60	15.5	51	79	27.2	28.4	0.001	9.6	0.047
TS9: complex risk of violence	5	6	2.2	33	37	8.0	61	49	6.1	128	59	6.9	52	67	4.8	38.4	0.001	30.9	0.001
TS10: institutional behavior	29	57	15.2	94	54	7.8	95	43	8.9	37	98	26.8	24	64	6.9	11.9	0.018	13.7	0.008
TS11: legal process	0	–	–	2	3	–	0	–	–	64	49	12.5	213	57	8.4	9.0	0.015	0.2	0.7

Calculated from Kaplan-Meyer survival analysis. Medians, standard errors and Log rank Mantel-Cox *X* ^2^, df = 4, *n* = 279 all patients; *n* = 187 those for whom a HCR-20 was available

### Scores on the DUNDRUM-2 triage urgency scale and length of stay

During the observation period, only the first item of the DUNDRUM-2 triage urgency scale predicted length of stay (Table 5). This remained true for the 187 for whom a HCR-20 was available (log rank Mantel Cox *X*^2^ = 25.5, df = 4, *p* < 0.001)

**Table 5:** DUNDRUM-2 triage urgency scale items and length of stay to discharge or end of study

Score	0			1			2			3			4			Log rank Mantel-Cox	
Item	*N*	Median	SE	*n*	Median	SE	*n*	Median	SE	*n*	Median	SE	*n*	Median	SE	*X* ^2^	*P*
TU1: location	1	11	–	24	24	4.9	118	55	6.7	63	76	22.3	73	61	20.4	25.5	0.001
TU2: mental health needs	8	46	65.5	55	54	11.1	127	61	5.6	83	53	6.1	8	28	14.1	2.9	0.570
TU3: suicide prevention	79	56	11.9	112	61	6.2	37	57	7.9	35	49	6.5	15	40	7.1	3.3	0.506
TU4: humanitarian need	181	57	5.7	8	102	61.5	8	62	284.3	59	49	10.9	22	48	15.2	4.2	0.381
TU5: systemic factors	7	20	11.8	11	26	36.9	8	22	14.9	20	49	31.3	232	57	4.9	7.1	0.131
TU6: legal urgency	138	53	3.5	57	61	9.2	45	97	53.7	9	30	13.4	29	46	14.7	5.5	0.239

Calculated from Kaplan-Meyer survival analysis. Medians, standard errors and Log rank Mantel-Cox *X* ^2^, df = 4, *n* = 279

### Risk assessment and length of stay

Table 6 shows that the HCR-20 item H1, ‘past violence’ predicted longer length of stay, as did item H2 ‘young age at first violence incident’. Item H9 ‘personality disorder’ predicted shorter lengths of stay. None of the remaining items predicted length of stay. Item C2 ‘negative attitudes’ also appeared to predict shorter length of stay.

**Table 6:** Historical-Clinical-Risk-20 (HCR-20 V2) scale items and length of stay to discharge or end of study

SCORE	0			1			2			Log rank Mantel-Cox	
Item	*N*	Median	SE	*n*	Median	SE	*n*	Median	SE	*X* ^2^	*p*
H1: previous violence	14	14	1.9	32	45	10.6	194	75	8.6	22.9	0.001
H2: young age at first violent incident	36	48	4.8	105	79	16.8	96	61	5.9	8.4	0.015
H3: relationship instability	23	59	25.6	50	69	4.7	166	64	7.1	1.6	0.443
H4: employment problems	27	118	84.8	49	76	6.9	164	60	5.4	4.0	0.134
H5: substance misuse problems	37	60	31.6	17	150	169.4	184	64	4.5	6.2	0.044
H6: major mental illness	17	46	24.7	26	14	6.4	197	71	5.2	14.4	0.001
H7: psychopathy (OMITTED)	–	–	–	–	–	–	–	–	–	–	–
H8: early maladjustment	48	55	4.0	62	85	27.1	124	64	6.4	2.4	0.306
H9: personality disorder	71	102	44.2	73	66	9.0	82	49	6.3	17.9	0.001
H10: prior supervision failure	52	59	7.2	37	147	60.8	150	64	6.1	2.5	0.282
C1: lack of insight	11	53	24.8	58	57	5.1	169	67	6.1	1.1	0.577
C2: negative attitudes	81	64	9.9	63	76	14.7	96	57	8.8	7.4	0.024
C3: active symptoms of major mental illness	44	48	6.6	59	58	5.5	137	72	9.4	3.7	0.160
C4: impulsivity	96	75	15.2	57	67	9.7	87	56	5.9	3.2	0.192
C5: unresponsiveness to treatment	33	53	4.6	103	67	5.1	103	76	15.2	4.3	0.115
R1: plans lack feasibility	24	64	13.5	39	54	3.1	176	70	6.6	2.1	0.353
R2: exposure to destabilisers	3	46	12.2	17	41	18.5	219	69	6.2	2.9	0.227
R3: lack of personal support	20	59	13.4	73	62	3.4	146	70	8.2	0.15	0.929
R4: non-compliance with remediation attempts	15	46	27.7	30	67	4.8	194	64	6.1	0.25	0.884
R5: stress	4	21	16.0	18	55	8.5	217	67	5.1	1.04	0.596

Calculated from Kaplan-Meyer survival analysis. Medians, standard errors and Log rank Mantel-Cox *X* ^2^, df = 2, *n* = 187

The DUNDRUM-1 11 item scale did not predict violence (AUC = 0.531, 95 % CI 0.450–0.611, *p* = 0.451) or seclusion (AUC = 0.498, 955 CI 0.425–0.571, *p* = 0.952); nor did the DUNDRUM-1 nine item scale predict violence (AUC = 0.539, 95 % CI 0.457–0.620, *p* = 0.341) or seclusion (AUC = 0.519, 95 % CI 0.466–0.611, *p* = 0.293). The DUNDRUM-2 triage urgency scale, a dynamic measure of need, also did not predict violence (AUC = 0.566, 95 % CI 0.485–0.646, *p* = 0.105) or seclusion (AUC = 0.539, 95 % CI 0.466–0.611, *p* = 0.293).

The HCR-20 total score predicted violence (AUC = 0.674, 95 % CI 0.598–0.751, *p* < 0.001) and seclusion (AUC = 0.694, 95 % CI 0.623–0.764, *p* < 0.001).

### Modelling predictors of length of stay

Using Cox regression to model the effect of the DUNDRUM-1-9 item scale on length of stay, with age and gender also entered as covariates, the omnibus test indicates a significant model *X*^2^ = 33.7, df = 3, *p* < 0.001. Gender did not have a significant effect though age did Wald *X*^2^ = 6.2, df = 1, *p* = 0.013, Exp(B) = 0.984 (95 % confidence interval of Exp(B) 0.972–0.997). The DUNDRUM-1 9 item score (scale range 0–36) had the largest effect on the model Wald *X*^2^ = 24.1, df = 1, *p* < 0.001, Exp(B) = 0.941, 95 % CI 0.919–0.964. If the mean score for the DUNDRUM-1 9 item scale (scale range 0–4) is entered instead of the total scale score, Exp(B) = 0.581, 95 % CI 0.468–0.722.

Using Cox regression to model the effect of the HCR-20 total score (scale range 0–40) on length of stay, with age and gender also as covariates, the model yielded is not significant (omnibus test *X*^2^ = 2.962, df = 3, *p* = 0.398) and no individual covariate approached significance (HCR-20 total score Wald *X*^2^ = 0.359, df = 1, *p* = 0.549).

When a model is constructed for all the variables of interest - age, gender, DUNDRUM-1 9 item score, HCR-20 total score (*n* = 187), main diagnosis and predicted legal status, the model converges at the third step (omnibus test *X*^2^ = 94.5, df = 7, *p* < 0.001). Age, gender and HCR-20 total score are not included. For the DUNDRUM-1-9 item score Wald *X*^2^ = 4.2, df = 1, *p* = 0.04, Exp(B) = 0.967, 95 % CI 0.936–0.998; main diagnosis on admission is significant only for the contrast of adjustment disorder with all other diagnoses Wald *X*^2^ = 6.9, df = 1, *p* = 0.009, Exp(B) = 3.843, 95 % CI 1.402–10.530; predicted legal status is significant only for ‘sentence’ contrasted with all other outcomes Wald *X*^2^ = 12.3, df = 1, *p* < 0.001, Exp(B) = 6.209, 95 % CI 2.240–17.205.

Repeating this model while omitting the HCR-20 in order to include all cases (*n* = 279), the model again converges at the third step. The omnibus test of the model is significant (*X*^2^ = 132.2, df = 7, *p* < 0.001). The DUNDRUM-1 9 item score (scale range 0–36) has Wald *X*2 = 16.1, df = 1, *p* < 0.001, Exp(B) = 0.951, 95 % CI 0.928–0.979, main diagnosis (adjustment disorder v all others) Wald *X*^2^ = 6.2, df = 1, *p* = 0.013, Exp(B) = 2.412, 95 % CI 1.205–4.828 and predicted legal status (sentenced v all others) Exp(B) = 6.914, 95 % CI 2.516–19.003. Substituting the mean DUNDRUM-1-9 item score (dividing the total score by 9 items to yield a range of 0–4) Exp(B) = 0.638, 95 % CI 0.513–0.795.

## Discussion

### Main findings

The patients admitted to this secure forensic hospital have the characteristics of medium secure forensic patients in other jurisdictions [61]. The start of this study coincided with a legal change that enabled the conditional discharge of patients found unfit to stand trial or not guilty by reason of insanity. There were no other legal or organizational changes during the period of the study so that the assumptions of survival analysis were not violated.

In this study we found that male gender predicted longer length of stay, while having no mental disorder other than an adjustment disorder at admission predicted shorter length of stay. Other diagnoses (schizophrenia spectrum disorders, affective disorders, organic disorders) had no significantly different effects on length of stay. Similarly legal status on admission and predicted legal status on admission predicted length of stay, but only in so far as a criminal justice disposal predicted shorter lengths of stay. Being found not guilty by reason of insanity or being detained under civil mental health legislation did not differ significantly - both tended to predict longer lengths of stay. Most items on the DUNDRUM-1 triage security scale predicted longer lengths of stay other than the suicide related items and the need to prevent access to weapons, drugs or media. The first item of the DUNDRUM-2 triage urgency scale concerning location as rated at the time of pre-admission assessment, predicted length of stay in a secure forensic hospital setting but other pre-admission indicators of urgency were poor predictors of length of stay. We found that higher scores on triage security item one ‘seriousness of previous violence’, triage security item three ‘immediacy of the risk of serious violence’, triage security item five ‘specialist forensic need’, triage security item six ‘history of absconding or eloping’, triage security item eight ‘victim sensitivity and public confidence issues’, triage security item nine ‘complex risk of violence’, triage security item 10 ‘institutional behaviour’ and triage security item 11 ‘legal process’ all predicted longer lengths of stay in the forensic hospital setting.

We found that events during the course of admission, including harm to self and violence towards others did not predict length of stay. Most episodes of violence did not lead to seclusion however if a patient required management in seclusion during the course of admission this did predict a longer length of stay.

These results resemble a Swedish prospective study of length of stay for forensic patients in which violent crime, legal status (restrictions on discharge), psychosis, substance misuse and absconding all predicted longer lengths of stay [46].

### Limitations

It was not possible to obtain a HCR-20 rating during the first 2 weeks of admission for 92 (33 %) of the patients admitted. This must have introduced a degree of bias, since those who could not be assessed had shorter lengths of stay and lower scores on the DUNDRUM-1 scale. Because a third of all patients admitted could not have a HCR-20 assessment completed in the first 2 weeks of admission, the failure of most of the HCR-20 items to predict length of stay may arise from the partial exclusion of short stay and female patients. 

However the same DUNDRUM-1 items remained significant predictors of length of stay for the 187 patients who did have a HCR-20 assessment (Table 3). For the 187 assessed, the HCR-20 appeared not to predict length of stay although the first item H1 ‘previous violence’ which conflates frequency and seriousness of violence, did predict longer length of stay as did H2 ‘young age at first violent incident’, H5 ‘substance misuse problems’ and H6 ‘major mental illness’. H9 ‘personality disorder’ predicted shorter length of stay, as did C2 ‘negative attitudes’. This curious finding is probably confounded by the finding that those who received a custodial sentence or other criminal justice disposal and those who had no mental illness other than an adjustment reaction had much shorter lengths of stay. In Ireland, mental health legislation excludes personality disorder from the legal category of mental disorder so that patients can only be found unfit to stand trial, not guilty by reason of insanity or detained under civil mental health legislation if they also have a mental illness or organic mental disorder [58].

Our findings therefore support the view that preadmission assessment can be used to predict length of stay in a secure forensic hospital.

The main limitation of this study was that patients had unequal follow up time. This occurred because of the prospective design of the study, which meant that patients were added to the database as they were admitted to the medium secure hospital and so for example a patient admitted at the beginning of the study in January 2010 had over 5 years of follow up time whereas a patient admitted in June 2014 had a shorter follow up time. In order to manage this we followed up all patients for a further 12 months after the last patient was added to the database to ensure each patient had a minimum follow-up period of at least 12 months. The use of survival statistical analysis using censored data is a well-established method for such studies in other clinical domains [60]. The median lengths of stay cited for those found UTP or NGRI are clearly under-estimates of the eventual completed lengths of stay. However a follow-up period in excess of 10 years would be required to ensure that all admissions had progressed to conditional or absolute discharge. This would impede access to valuable clinical knowledge and service planning. Also, by the time all have been discharged there will probably be further revisions of legal frameworks, health service arrangements and advances in treatment, changes that would violate the assumptions^1,2^ of the statistical models [41, 60]. The prospective design of the study also had a number of advantages. For example, as the DUNDRUM-1 and DUNDRUM-2 scores were completed prior to admission, this eliminated the risk of bias whereby raters might have been inclined to rate patients differently if they had proved difficult to manage in the secure hospital setting after admission. On balance, we were of the view that the advantages of a prospective design outweighed the potential limitation of unequal follow up time.

### Interpretation

One of the greatest risks facing a patient who is admitted to a secure forensic hospital is the risk of a long stay. It is unacceptable both from a human rights and economic perspectives to detain patients at a level of therapeutic security higher than they require. However moving patients to lower levels of therapeutic security prematurely or discharging patients to the community when they are not yet ready for such a move would place patients, staff members and the public at risk of serious harm. Therefore length of stay in forensic settings is a crucial issue for patients, clinicians, managers and service commissioners. Currently, decision making regarding risk assessment in forensic psychiatry has evolved to the use of structured professional judgment instruments. We have shown in this study that selected items on the DUNDRUM-1 triage security scale and DUNDRUM-2 triage urgency scale, rated at time of pre-admission assessment, predicted length of stay in a medium secure forensic hospital setting. We think this evidence may form the basis for developing a new structured professional judgment instrument which could be rated prior to or at the time of admission in order to estimate length of stay for newly admitted forensic patients. We believe such a tool would be invaluable to clinicians, service planners and commissioners given the expensive nature of providing secure care.

### Generalisability

In a meta-analysis of unstructured clinical judgement across a range of domains of professional opinion concerning various types of risks, predicting the length of psychiatric hospital admission was found to be no better than chance [62]. This research study examined factors predicting length of stay in the secure forensic hospital for the Republic of Ireland. We found that items on the DUNDRUM-1 triage security scale rated prior to admission predicted length of stay and were better predictors of length of stay than events during the course of admission. Given that high secure units in many jurisdictions were designed as long-stay units, medium secure units as medium-stay units and low secure units as short-stay units and that these tools were designed to assist decision making concerning the need for therapeutic security, it is not surprising that a tool which was designed to separate patients according to the level of therapeutic security needed also has predictive power for length of stay. The DUNDRUM-1 triage security scale is now being used in another jurisdiction and has translated well to that jurisdiction, as an aid to pre-admission decision-making [61]. We think scores on the DUNDRUM-1 triage security scale may also predict length of stay in other jurisdictions.

The absence of any effect of an initial assessment of risk on length of stay merits some comment. Only two thirds of admissions could be rated on the HCR-20 in the first 2 weeks of admission. While this study does not therefore exclude the possibility that a risk assessment might contribute to the prediction of length of stay, clinicians appear to be reluctant to complete a HCR-20 in the earliest stages of admission.

Those with relatively shorter lengths of stay may arise from the high proportion of patients admitted either on remand (pre-trial) or following sentence who were not made subject to a mental health disposal. As a result, many of those thought likely to be unfit to stand trial or NGRI were instead given fixed sentences and released at fixed times irrespective of risk. Although some were diverted to local mental health services [63], many were not. This may require legal reform in this jurisdiction.

### Future research

In a systematic review of factors affecting outcome for older medical patients, cognitive ability was judged to be a key determinant of length of stay [64]. Because cognitive impairment is associated with a range of psychiatric disorders it may also account for length of stay within forensic hospitals [65]. Recently we found that a nationally representative cohort of forensic patients scored more than three standard deviations below the mean on the MATRICS Consensus Cognitive Battery [66]. But to date, the role played by cognition in determining length of stay within general psychiatric services, let alone forensic services has scarcely been studied. However the few studies which have been conducted are promising [67–69].

In one study where a cognitive assessment was completed within 72 hours of admission, two cognitive variables accounted for more variance in length of stay than demographic factors and clinical domains [68]. The two tests were Trails A, and Visual Reproduction taken from the Wechsler Memory Scale, where each accounted for 21.6 and 16.3 % of the outcome variance respectively. A regression model combining both of these variables with the Mini-Mental State Examination (MMSE) and patient diagnosis predicted 82 % of patients who had a length of stay greater than 21 days.

Cognitive impairments may lead to increased length of stay within psychiatric services in a variety of ways. First, meta-analytic studies have demonstrated that cognition is a strong contributor to ‘real world’ functional outcomes [70]. Second, cognitive deficits may underpin symptoms especially those of the negative and disorganised type and therefore limit the efficacy of pharmacotherapy [71–73]. Third, within forensic services cognitive deficits may interfere with patient’s ability to benefit from treatment programmes [4, 5] for example by impairing self-monitoring and insight [9]. Understanding if and how cognitive deficits contribute to length of stay as well as interfere with recovery is therefore an important research priority.

We plan to assess the extent to which measures of clinical need for therapeutic security such as the DUNDRUM-1 and routine measures of outcome in a forensic setting such as the DUNDRUM-3 programme completion scale and DUNDRUM-4 recovery scale are influenced by neurocognitive ability. This would provide a possible explanation for the relationship of the DUNDRUM-1 to length of stay.

## Conclusions

The DUNDRUM-1 triage security scale was designed to assess need for therapeutic security. Items from this scale, together with diagnosis at the time of admission (adjustment disorder) and predicted legal status may be used to predict length of stay in a secure forensic hospital. The need for seclusion during the course of admission is also a useful potential predictor. Future research into factors predicting length of stay will consist of combining those items in order to develop a new structured professional judgment instrument to assist in predicting likely length of stay in secure forensic settings.

## Footnotes

1 When the second event (discharge) has not yet occurred for some cases, these cases are said to be ‘censored’. The Kaplan-Meyer model allows comparison of survival rates between two or more groups. The model assumes that probability of discharge depends only on time since admission. The probabilities of hazard (discharge) are assumed to be stable with respect to absolute time, so that cases admitted at different times should behave similarly. There should also be no systematic difference between censored and uncensored cases. Factors known or determined at the time of admission can be used to assess group differences. The equality of the survival distributions of different cases of a given factor can be tested using the log-rank test if all time points are weighted equally and this assumption is adopted here.

2 If the event (discharge) has not occurred, the case is said to be censored. Censored cases are not used in the computation of the regression coefficients, but are used to compute the baseline hazard. The proportional hazards model adopted here assumes that the covariates act in a constant way over time, and do not vary over time. The proportional hazards model assumes that the time to discharge is described by a hazard function, which is a measure of the potential for discharge to occur at a particular time, given that the event did not yet occur. Larger values of the hazard function indicate greater potential for the event to occur i.e. that length of stay will be shorter. The Exp(B) statistic describes the likelihood of hazard (discharge) as time progresses, compared to another group or for a continuous variable, the increase or decrease for each unit change in the variable. A value of Exp(B) less than zero e.g. 0.950 means that the hazard of discharge is reduced by 100 % − (100 × 0.950) for one unit increase in the variable, and a five point increase in the variable would mean that the hazard of discharge is decreased by 100 − (100 × 0.950^5^) = 22.6 %. The length of time to discharge would be increased and can be calculated from the model. Conversely, a value of Exp(B) greater than 1 would increase the hazard of discharge and would shorten the length of time to discharge.
